# Active Parents–Active Children—A Study among Families with Children and Adolescents with Down Syndrome

**DOI:** 10.3390/ijerph18020660

**Published:** 2021-01-14

**Authors:** Ann-Christin Sollerhed, Gerth Hedov

**Affiliations:** 1 Faculty of Teacher Education, Kristianstad University, Elmetorpsvägen 15, 291 39 Kristianstad, Sweden; 2 Faculty of Health Sciences, Kristianstad University, Elmetorpsvägen 15, 291 39 Kristianstad, Sweden; gerth.hedov@hkr.se

**Keywords:** physical activity, adolescents, children, Down syndrome, sports activities, parents, siblings

## Abstract

From a public health perspective, it is important that children with Down syndrome (DS) lay the foundations of physical activity (PA) early in life to keep active in school, as teenagers and as adults. The aims were to investigate PA patterns in children and adolescents with DS, as well as their parents’ and siblings’ PA patterns. Methods: A survey was performed among 310 families with children with DS (54% boys and 46% girls) aged 8–18 years (mean 14.04, SD 3.18) in Sweden. Chi-squared tests and multiple logistic regression were carried out. Results: Nineteen percent of children and adolescents with DS and 34% of the parents were active three or more times per week. The child’s PA level was significantly associated with parents’ PA (OR = 5.5), siblings’ PA (OR = 5.1) and the child’s locomotion ability (OR = 3.5). Physically active parents had active children to a greater extent than inactive parents (59% vs. 29%; *p* < 0.001). Conclusions: Physically active parents have active children. To promote PA among children and adolescents with DS, it is important to promote and pay attention to the parents’ and siblings’ PA behavior, as children with DS are dependent on support from the family.

## 1. Introduction

Down syndrome (DS) is the most common genetic cause of intellectual disability (ID). The physiological characteristics associated with DS can impact an individual’s ability to participate in physical activity (PA). Despite difficulties being active, it is important that children with DS are physically active as the long-term consequences of their inactivity can lead to serious health problems. It is important to understand and design appropriate interventions for this group of children. DS accounts for 8% of all registered cases of congenital anomalies in Europe. The overall prevalence of DS in the world is 10 per 10,000 live births. In recent years, the prevalence of DS has been increasing, though it varies between different countries [1].

DS is a genetic condition that causes delays in both physical and intellectual development. A non-disjunction and division of cells have taken place. Instead of having the typical two copies of the 21st chromosome, people with DS have three copies. Physical and mental disabilities vary in terms of severity. A set of anatomical, physiological, cognitive and psycho-social attributes predispose them to limitations regarding PA and fitness [2]. Low muscle tone, skeletal issues and ligament laxity cause instability in joints. Difficulties in initiating movements, difficulty with equilibrium and balance and postural control affect the movement skills and ability to be physically active [2]. Children with DS experience significant delays in the onset of motor milestones and display quantitative and qualitative differences in movement patterns compared to children with typical development. They have a reduced PA ability compared to other children, as well as compared with other mentally disabled children [3].

It is important that children with DS develop routines for PA and exercise early in life to establish a healthy lifestyle and to maintain a life-long functional level of physical fitness [4], especially given that individuals with DS are living longer than ever before [5]. PA confers benefits to both physical and psychosocial health, as well as the functional ability and quality of life [6,7,8]. There is convincing evidence that PA reduces or delays the onset of most kinds of diseases [9]. PA early in life is important to present and future health [10,11,12]. PA provides multiple benefits in children’s and adolescents’ health while physical inactivity is a serious threat to the health and wellbeing [13,14,15,16]. Nevertheless, time for PA decreases and time for inactivity and sedentary behavior increases in young people in general [17] but the inactive proportion is higher among young people with DS than in the rest of the population [18,19,20,21,22,23], especially in high-intensity activities [24].

Children and adolescents in general sit more, walk less frequently and do less PA than children did some decades ago [13,25,26]. It has been estimated that 80% of young people, 11–17 years old, do not reach the minimum recommended 60 min of moderate to vigorous PA (MVPA) per day [17]. The proportion of children with DS not reaching the lowest level of recommended daily MVPA is higher than among other children [2,27] and they become less active and more sedentary as they become older [28]. Sedentary behavior is characterized by an energy expenditure ≤1.5 metabolic equivalents while in a sitting, reclining or lying posture [29]. Sitting in front of a screen, game play and transportation with cars contribute to increased sedentary time among children and adolescents [30], which is a threat to future public health [31,32]. Sedentary behavior is associated with an increased risk of all-cause mortality and various physiological and psychological problems [16,33,34,35], also in children [36]. It is alarming that children’s PA levels are low, given that physical inactivity and sedentary behavior have been found to be associated with lower fitness and diverse unhealthy outcomes, which affect health outcomes later in life [37,38,39,40,41,42].

As there are few studies addressing PA among children with DS and, to our knowledge, no such studies carried out in Sweden, the purpose of this study was to investigate PA patterns in children and adolescents with DS, as well as to investigate their parents’ and siblings’ PA patterns.

## 2. Materials and Methods

### 2.1. Design and Sample

The study design was a cross-sectional survey. The study included 310 children and adolescents with DS (168 boys, 132 girls) between 8 and 18 years old; the average age was 14.04 years (SD = 3.18). The inclusion criteria for participation in the study was families with children with diagnosed DS in the age of 8–18 years. The participating families came from all parts of Sweden, from north to south, urban and rural areas. Forty-three percent of the participating families lived in cities, 37% in small villages and 20% in the countryside. The habitation was 71% in single-family homes, while 19% lived in apartment blocks and 10% on farms or in cottages (*n* = 310). More than half of the children (56%) had siblings, 32% of the older and 24% younger full siblings, 20% had older and 1% had younger half-siblings. Thirty-two percent of the children with DS had one sibling, 24% had two, 24% had three and 20% had four siblings. Data were obtained using a questionnaire completed by the parents. All information about the children with DS and their siblings was received from their parents. Thus, the sample consisted of 310 families. The families were recruited through state rehabilitation centers or by a non-profit membership-based association for children with DS, where information on the study was advertised on the associations’ website. The non-profit membership-based association for children with DS had about 3000 members at the time for the performance of the study. The parents who were interested in participating send an email to the researchers and received a letter with information and a personal invitation to participate. The response rate of questions in the survey ranged from 98% to 100%. The number of respondents for each question will be presented in parenthesis in the following text.

### 2.2. Ethics

The study was conducted according to the ethical principles for medical research involving human subjects and all procedures in the study were in accordance with the Declaration of Helsinki. The Research Ethics Committee approved the study at the Faculty of Medicine, Lund University (LU 2014/280).

### 2.3. Measures

The survey included 54 questions on demographics, PA patterns of the children and adolescents with DS, participation in any sports activities, the parents’ and any siblings’ PA patterns, the DS child’s sedentary behavior, the parents’ perceptions and experiences of barriers and facilitators for PA for the child with DS and the child’s ability to perform different movement skills. Table 1 summarizes the survey questions and the dichotomization of the response items used in the multiple linear regression analysis in this article.

In addition to the questions presented in Table 1, the other used questions are presented below.

“How important do you think PA/exercise is in general?” With four response items: “Very important; Quite important; Not very important; Not at all important.”

“Are you satisfied with the child’s PA level?” With four response items: “Yes, very satisfied; Yes, quite satisfied; No, not very satisfied; No, not at all satisfied.”

“Do you perceive that the child is satisfied with its own PA level?” with four response items: “Yes, very satisfied; Yes, quite satisfied; No, not very satisfied; No, not at all satisfied.”

“Does the child voluntarily want to do PA?” with four response items: “Yes, most of the time; Yes, quite often; No, not very often; No, never.”

The child’s involvement in PA of various intensities was assessed by the question: “To what extent does the child exercise, do sports- or outdoor activities in his or her leisure time in low, moderate or high-intensity PA for more than 20 min of activity at a time? Please assess the number of occasions per week in the three different intensity levels.” With five response items for each intensity: “Never; Very seldom; Once a week; Twice a week; Three times or more a week.”

The range of sports activities for the child with DS was assessed by the question: “Does the child participate in any sports activities every week in his/her free time during the season? If the child practices one or more sports regularly, please write which one or which ones.”

The parents were asked to assess the child’s motor skills, balance skills and endurance capacity in comparison with other children’s skills. “Do you experience that the child’s motor ability differs from other children’s? with nine response items: “No differences; Movements are slower; Balance is less good; Reaction time is slower; Endurance skills are lower, Fitness is lower; Movements are faster; Balance is better; Reaction time is faster; Endurance skills are higher; Fitness is higher.”

The child’s movement ability/inability was assessed by the question: “According to your experience, what type of movements/activities has the child problems to perform or to participate in?” with nine response items: Ball games together with other children; Ball games together with other children but with a personal helping adult; Swimming; Fast running in short distances; Running with changes of directions; Endurance activities; Cross movements where the centerline of the body is crossed; Fine motor skills such as threading beads, tying, embroidering.”

The child’s physical fitness was assessed by the question: “Do you experience that the child’s physical fitness differs from other children’s? with five response items: “Much better; Better; Neither better nor worse; Worse; Much worse.”

The parents were asked to assess if the DS child was physically active according to the World Health Organization (WHO) recommendations by the question: “Based on the WHO’s recommendation of at least 60 min of daily heart rate-enhancing PA (MVPA), do you feel that your child is achieving this?” With four response items: “Yes, most often; Yes, quite often; No not often; No never.”

The child’s sedentary behavior was assessed by the question: “Do you think that your child is more sedentary and sits still more compared to other children, also compared to children without disabilities?” With five response items: “Much more, More, Neither more nor less, Less, Much less.”

### 2.4. Statistical Analysis

Statistical analyses were performed using the statistical package IBM SPSS Statistics for Windows Version 23.0 (IBM Corp., Armonk, NY, USA). First, descriptive statistics with frequencies were used to summarize the characteristics of the cohort. Second, the Chi-square test was used to determine the significance of differences when comparing groups of children and adolescents. Third, a multiple linear regression was carried out using a logistic regression model (method: enter) with the DS children’s PA as the dependent variable. Independent variables included in the model were gender, parents’ PA, siblings’ PA, living region, child’s locomotion ability, transportation to activities, child’s membership in a sports club, parents’ membership in a sports club, child’s willingness to exercise and child’s involvement in high-intensity PA (Table 1). The Hosmer and Lemeshow goodness-of-fit test and the Nagelkerke R^2^ test [43] were used to measure the quality of the regression model. The significance level was set at *p* < 0.05. Adjusted estimates and their 95% confidence intervals, as well as the *p*-value, were used.

## 3. Results

A total of 7% of the children and adolescents with DS (*n* = 307) had regular physiotherapy every week and 18% had regular special training in school or daycare. All the children and adolescents had physical education (PE) in school; 26% once a week, 59% had PE lessons twice a week and 12% three times a week. About 99% of the parents thought that PA was very or quite important for health. Endurance activities, changing directions in locomotion, sprint and swimming were perceived difficult to manage for the children with DS. A summarize of the parents’ perceptions of the children’s difficulties, health, PA patterns, sedentary behavior, fitness and physical abilities are shown in Table 2. 

About 69% of the children with DS were reported to be active in any sports activity (*n* = 308) and a wide variety of sports was mentioned: football, swimming, floor hockey, dancing, ice hockey, riding, rugby, archery, wrestling, karate, running, cycling, gymnastics, orienteering, bowling, spinning, tennis, cross-fit, alpine skiing, cross country skiing, track and field, volleyball, handball, basketball, yoga, climbing, diving, parkour, water aerobics, badminton, table tennis, golf, canoeing, motocross, skateboard, biathlon, boxing, aerobics and cheerleading.

A total of 28% of the children and 30% of the parents were physically inactive. About half of the children were reported to be physically active once or twice per week. A total of 19% (*n* = 308) of the children and adolescents with DS were reported to be physically active, so they became sweaty and short of breath (at least half an hour three times or more per week), while the amount of physically active parents was 34% (Table 3. The parents were also asked to assess how often the child with DS was involved in PA with low, moderate or high intensity. About 21% were reported to be active in PA with high intensity at least twice a week, 24% in moderate-intensity and 42% in low intensity for at least 20 min sessions (Table 3).

A correlation between parent and child PA level was found. The physically active parents had active children to a greater extent than inactive parents had (59% vs. 29%; *p* < 0.001).

The parents assessed the children’s and adolescents’ ability to walk, jog or run the distance of 3 km. About 10% of the children and adolescents with DS were reported not to be able to walk 3 km at all, 24% could walk the distance if they could stop and rest, 38% could walk the distance without stops, 20% could walk at a moderate speed, 6% could jog the distance slowly and 2% could run at a moderate speed (*n* = 310). The children’s ability to move the distance 3 km in relation to age groups are shown in Table 5. PA patterns and sedentary behavior in relation to age groups are also shown in Table 4. 

The multivariate logistic regression analysis results with the DS child’s PA as the dependent variable are shown in Table 5. Variables significantly associated with the children’s PA were in order: the parents’ PA (OR = 5.5), the siblings’ PA (OR = 5.0) and the child’s locomotion ability (OR = 3.5).

## 4. Discussion

The study’s main goal was to investigate PA behaviors among children and adolescents with DS through their parents’ perceptions and experiences. The children’s PA was found to be associated with their parents’ and their siblings’ PA and the child’s own locomotion ability.

In our study, a correlation between parent and child PA behavior was found. Physically active parents had active children to a greater extent than more inactive parents. The children’s PA levels were associated with both their parents’ and their siblings’ PA levels, which could be interpreted as meaning that PA was a family affair and included both parents and children. Child PA levels and parental support are strongly correlated for all children [44,45,46]. However, it could be even more pronounced for children with DS because they are more dependent on guidance and support, even at older ages. The family setting is important in determining how much PA children with DS undertake [3]. Parents serve as role models for their children; they help the children with practical requisites to engage in activities, give mental encouragement and pay attention to improvements. Enjoyment is strongly associated with motivation and the balance between skills (competence) and the challenge is vital to experience enjoyment. Adjusting the challenge for these children by taking into consideration the difficulties the diagnosis causes is extremely important. However, the activities should be challenging and not be so easy that the children feel bored. Parents and siblings are important motivators and family modeling is frequently reported as an important correlate for life long PA [47] and early participation in PA is associated with a greater likelihood of involvement later in life [47,48,49]. The parents of children with DS need support from PE teachers and sports associations about the best practice for their children and adolescents.

The parents influence all siblings in the family but siblings also affect each other. The siblings of children with DS often assist and help to motivate and inspire their sibling with DS. The siblings’ interest in PA becomes a reinforced strong effect for PA in the family setting, which might originate from the parents. Still, the influence of the sibling is in itself a strongly associated factor for the PA for the child with DS. Children who have a brother or sister with DS are often expected to be responsible for their sibling’s well-being once their parents are no longer able to do so [50] and the engagement starts early. The siblings, both older and younger, become important role models for their brother or sister during childhood. Thus, it is vital to involve the siblings in interventions for increased PA for children with DS, without placing too much responsibility on the sibling. It is a balancing act, where the sibling must be able to carry out their own activities and activities together with the sibling with DS.

In our study, almost all parents (99%) stated that PA was important for health; however, only a third of the parents were physically active themselves. There are likely many reasons why the parents were not active though they thought PA was important; we did not investigate the reasons. Most parents had positive attitudes towards PA; however, the transmission of attitudes and habits from parent to a child became more efficient when it comes to parents’ actions. Children do not do as parents tell the children to do; children do as parents do. The children are subjects to the transmission of parental beliefs and values towards PA. The transmission from parents might be even more important for children and adolescents with DS as they are less attached to peers and more referred to as their parents and siblings. Sometimes the DS children are at risk of becoming socially isolated as they, compared to their mainstream peers, show less frequent peer interaction [51]. Children and adolescents with DS need both mental and practical parental support to a large extent. The parental support is decisive high up in age, which requires more perseverance from the parents and sometimes also from siblings. The family setting means both facilitators and barriers to the children’s PA participation [46,52].

According to Barr and Shields (2011), the family setting was number one as a facilitator and number two as a barrier for PA [3]. Thus, both positive and negative outcomes from the family setting. The motor learning process and the social decoding process is both much longer and more complicated for the children with DS [53] and puts the parents in a sustainability project where they have to give constant support and encouragement.

Though the influence of parents’ behavior on children’s behavior is strong, the child’s physical ability is decisive for the PA patterns. In our study, the children’s locomotion ability was associated with the children’s PA level. It was also shown that children who were able to perform PA with high intensity were more active. Many sports have elements of high-intensity PA and endurance, which limit the possibility for children and adolescents with DS to participate [24], as a set of anatomical attributes limits PA and fitness [2]. However, early training of infant and toddler movements has been shown to impact fitness and motor skills among these children [54]. It is vital to start motor training early in life for all children. For children with DS, this is perhaps even more important as they are not as self-functioning as other children and all motor learning takes more time for these children. Even if it takes more time, they should be encouraged to continue. PA in the form of self-generated actions and exploratory motor behaviors early in life is important for proper overall development among children with DS [55] but also external motivation from significant people in the child’s vicinity. Parents’ probability of supporting early life PA and motor behaviors for their children is higher if the parents are interested in PA themselves and have positive attitudes towards it. Infants with DS who received high-intensity PA training early in life were found to benefit from this training [56], which again emphasizes the parents’ role for PA.

The locomotion skills were assessed via a question about the child’s ability to walk, jog or run the distance of 3 km. About 10% of the children and adolescents with DS could not walk the distance at all and a quarter had difficulties doing so. Less than 10% could jog or run the distance slowly and about 80% could walk at different speeds with or without stops. Thus, many of the children and adolescents had difficulties moving a distance, especially when it came to jogging or running. A significant difference in ability to move the distance was seen in relation to the age of the children. A higher percentage of the older children was able to move the distance than the younger. The ability to move the distance is dependent of the development of motor competence. The development of motor competence during childhood is dependent by the growth and maturity characteristics of the child interacting with the environment such as opportunities and restraints for movement, which interact with the biological substrates of growth and maturation to determine the mobility capacity of the child [57].

Children with DS have lower running performance than mainstream peers but also compared with children with other disabilities [58]. Difficulties with walking/running performance, together with low endurance capacity, strongly influence and affect the number of activities the children can participate in. However, some children and adolescents in our study were reported to be very active in a wide variety of sports, also in sports with high claims of locomotion, motor control and endurance. These sports included climbing, canoeing, boxing, archery, skiing, parkour and biathlon. The parents’ special sports interests are mirrored in their children. Familial and other environmental factors have an influence on the children’s behavior, such as involvement in different sports [59]. Some sports are expensive to perform and costs for equipment and transportation can also be a burden and reliant on the family’s economic resources [60]. We did not investigate the financial situation of the families but are aware of the importance.

The parents were asked to assess the number of PA occasions the children with DS were involved in three different intensity levels: low, moderate and high intensity. A small proportion of the children, about 8%, was active in high-intensity PA three times or more a week and 7% in moderate intensity. Thus, less than a fifth of the children were active in moderate to vigorous PA (MVPA) three times or more a week. We asked about MVPA sessions for at least 20 min, which is far less than the recommended daily amount of PA [32]. According to the parents’ answers about whether they thought that the children reached the recommended level of daily PA, the result showed that one-third of the parents thought that the children met the recommended level MVPA. The parents might overestimate the children’s daily MVPA as only 10–15% were reported to be active in MVPA for 20 min, which matches the results of other studies [2,27,61].

Physical inactivity and sedentary behavior are a serious threat to health and wellbeing [13,16,26] and it is vital to promote PA also among children with DS. It is alarming that inactivity and sedentary behavior increase among children and adolescents and there is a call for action. One way could be to engage parents and whole families in promotion for PA.

### Strengths and Limitations

The study design was cross-sectional, which limits conclusions about causality. The children’s, siblings’ and parents’ PA were reported and not measured, which is a limitation. A combination of self-reported and measured PA by accelerometers would have been optimal [62,63]. However, we did not have the possibility to use accelerometers. We suspect that the PA levels are partially overestimated by the participants in our study, especially when it comes to the reported vigorous PA and the achievement of the 60 min daily MVPA. The questionnaires consisted of single-item questions, which is a limitation. However, the questions in the questionnaire have earlier been used and found to be reliable and valid among Swedish school-aged children and adolescents.

All variables included in the study were reported by the parents and could be affected by their perceptions. We recruited the families through a non-profit membership-based association for children with DS, where information on the study was advertised on the associations’ website and parents who wanted to attend contacted us. This could have caused a predominantly positive selection of families with parents positively committed to PA and the results might have a skewness to the positive side. The prevalence of inactive children with DS is probably higher, which has been shown in other studies [23,24]. In our sample, only 7% of the children had regular physiotherapy or special training in school or daycare, which could imply a positive sample from a disability perspective. Although the sample might be composed of a predominantly positive group of families, the results show that the PA level in this group of children and adolescents is too low and needs to be increased.

The study has strengths that are worth mentioning. The sample included more than 300 families with children with DS, which gives a good picture of PA habits for this group of children. To the best of our knowledge, there are very few, if any, research about PA among children and adolescents in Sweden. The questionnaire consisted of several questions about the PA patterns to get a picture of habits, amount of PA and intensity of PA among children and adolescents with DS. The results show the great importance of the family setting where the parents’ attitudes and actions are vital for the children’s PA behavior.

## 5. Conclusions

The interest, attitudes and habits of PA are carried over, from parent to child. To promote PA among children with DS, it is vital to pay attention to their parents’ and siblings’ PA behavior and to provide parents with support in their upbringing of physically active children. This is vital for all children and parents; however, even more, pronounced for children with DS because they are more dependent on guidance and support high up in age. Parents need support from sports coaches, child health care and physical education (PE) teachers and it is crucial that these categories have adequate knowledge about DS and facilitators and barriers for PA connected with DS. The benefits of PA for health are well known but the information about the PA patterns among DS children and adolescents is limited and many parents often feel incapable of how to increase PA and how to train the children’s fundamental movement skills. From a public health perspective, it is important to pay attention to PA in the whole family and not just the child with DS solely. Information on PA habits of children and adolescents with DS in Sweden is low and the perceptions of their parents would help to guide the development and design of needed support and strategies to promote PA in this group of children. This study contributes to the current knowledge of PA behavior in families with DS children. The number of children and adolescents with DS who accumulate periods of satisfactory PA levels is low. The study provides results that active children with DS have active parents and that the interest, habits and attitudes are carried over, from parent to child, as well as among children with DS.

## Figures and Tables

**Table 1:** Variables with corresponding questions from the survey, response options in the survey and dichotomization for statistical comparison between groups.

Variable	Response Options in Questions	Dichotomized
Gender Question: Are you?	Boy/Girl	Boy Girl
Physical activity (PA) in leisure-time (child with Down Syndrome (DS), parents, siblings) Question: How often do you exercise or train during your leisure time for a period of at least half an hour and get out of breath or sweaty?	Never A few times a year A few times a month Regularly, once a week Regularly, twice a week Regularly, three times a week Regularly, four or more times a week	PA Often (6–7) PA Seldom/Never (1–5)
Living Question: Do you live in …..?	In a city In a village In the countryside	In a city (1) In a rural environment (2–3)
Locomotion capacity Question: What do you think your child with DS can manage? Please mark next to the statement that best fits the child’s locomotion capacity.	Able to run 3 km in high speed Able to run 3 km at moderate speed Able to jog 3 km at low speed without stops Able to walk 3 km at moderate speed without stops Able to walk 3 km at low speed without stops Able to walk 3 km but with stops now and then Not able to walk 3 km	Ability to move the distance at least at moderate speed without stops (1–4) Inability to move the distance at least at moderate speed without stops (5–7)
Transportation to activities Question: If you think about the child’s activities and how they get to and from the activities. Does the child with DS usually walk or cycle or is the child transported by car or bus?	By car or bus Walk Bicycle	Car or bus (1) Walk or bike (2–3)
Member in a sports club (child with DS, a parent) Question: Is the child with DS a member of any sports club?	No/Yes	No Yes
Willingness to be physically active Question: Does the child want to go to physical activities voluntarily?	Yes, most often Yes, quite often No, seldom No, never	Yes (1–2) No (3–4)

**Table 2:** Parents’ perceptions of physical activity (PA), the child’s health, the child’s PA patterns, the child’s willingness to do PA, the child’s sedentary behavior and difficulties in PA for the child (%).

Parents Perceived That ……	Percentage of Parents (%) *n* = 310
PA is very important for health	76%
PA is quite important for health	23%
The child’s health is very good	46%
The child’s health is quite good	45%
The child’s PA patterns are satisfactory	59%
The child is satisfied with its own PA patterns	93%
The child spontaneously wants to do PA	65%
The child is more sedentary than children without disabilities	47%
The child is neither more nor less sedentary than children without disabilities	40%
The child is less sedentary than children without disabilities	13%
Endurance activities are difficult for the child to perform	90%
Changing directions in locomotion are difficult	68%
Sprint activities are difficult	62%
Swimming is difficult	47%
Playing ball games together with other children is difficult	81%
The child has poorer motor skills than children without disabilities	89%
The child has poorer balance skills than children without disabilities	86%
The child has poorer endurance capacity than children without disabilities	87%
The child has poorer fitness than children without disabilities	38%
The child has neither better nor poorer fitness than children without disabilities	32%
The child has better fitness than children without disabilities	30%
The child achieves WHO’s recommended daily 60 min moderate to Vvgorous physical activity (MVPA)	33%

**Table 3:** Frequency of physical activity (PA) sessions for at least half an hour sessions to get sweaty and short of breath among children with Down Syndrome (DS) and among parents. Frequency of PA in high, moderate and low intensity for at least 20 min sessions among children with DS (%).

Frequency of Physical Activity (PA) How Often?	Children’s PA (%)				Parents’ PA (%)
	PA sessions for at least 20 min (sweaty and short of breath) (*n* = 308)	PA in high intensity (*n* = 280)	PA in moderate intensity (*n* = 277)	PA in low intensity (*n* = 282)	PA sessions for at least 20 min (sweaty and short of breath) (*n* = 307)
Never	10	36	18	7	7
Seldom (few times a year/month)	18	20	24	19	23
Once or twice a week	53	36	51	57	36
Three times or more a week	19	8	7	17	34

**Table 4:** Locomotion capacity, physical activity (PA) in leisure-time, membership in sports clubs, sedentary behavior and parents’ PA in relation to age groups among children with Down Syndrome (%).

Variables *	Age 8–9 (%) *n* = 52	Age 10–12 (%) *n* = 82	Age 13–15 (%) *n* = 92	Age 16–18 (%) *n* = 82	*p*-Value **
**Locomotion capacity; distance 3 km**					
Able to run in high speed without stops	--	4	--	--	--
Able to run at moderate speed without stops	2	--	3	--	
Able to jog at low speed without stops	2	6	10	4	
Able to walk at moderate speed without stops	8	13	26	29	
Able to walk at low speed without stops	36	38	28	49	
Able to walk but with stops	38	29	23	11	
Not able to walk the distance	13	10	10	7	
**Locomotion capacity; distance 3 km, dichotomized**					
Ability to move the distance in at least moderate speed without stops	13	23	39	33	0.006
Ability to move the distance at low speed with stops/inability	87	77	61	67	
**Children’s physical activity (PA) in leisure-time**					
PA often (at least 20 min three times a week)	20	16	20	21	0.864
PA seldom or never (less than 20 min twice a week)	80	84	80	79	
**Member in sports clubs**					
Yes, member	41	50	55	61	0.525
No, not member	59	50	55	61	
**Sedentary behavior**					
Not sitting so often	69	62	48	38	0.001
Sitting often	31	38	52	62	
**PA among parents**					
PA often (at least 20 min three times a week)	35	31	40	33	0.673
PA seldom or never (less than 20 min twice a week)	59	50	55	61	

* Variables in bold ** *p*-value when statistical comparison between groups.

**Table 5:** Variables associated with physical activity (PA) among children/adolescents with DS. Results from a logistic regression model. Odds ratios (OR) and 95% confidence intervals (CI) and *p*-Value (*n* = 310).

Variables	Wald	OR	95% CI for OR	*p*-Value
Gender (male)	2.54	1.77	0.88–3.57	0.111
Parents’ PA	20.81	5.46	2.63–11.31	<0.001
Siblings’ PA	19.91	5.05	2.48–10.29	<0.001
Living–form of housing	0.22	1.18	0.57–2.37	0.643
Locomotion ability	9.36	3.50	1.60–7.63	0.002
Transportation to activity	2.49	2.28	0.82–6.32	0.115
Member in sports club, child	0.10	0.89	0.42–1.88	0.751
Member in sports club, parents	1.90	1.80	0.78–4.16	0.168
Willingness to do PA	0.16	0.95	0.42–2.15	0.899

Hosmer-Lemeshow, *p* = 0.007, Nagelkerke R^2^ = 0.438.

## Data Availability

New data were analyzed in this study. Data sharing is not applicable to this article.
